# Supplementary material for: Microtentacle Formation in Ovarian Carcinoma
Source: Cancers (Basel). 2022 Feb 4;14(3):800. doi: 10.3390/cancers14030800 (PMC8834106; doi:10.3390/cancers14030800)
Supplement: Supplementary file 1 [file cancers-14-00800-s001.zip › cancers-1455481-supplementary.pdf]

Figure 3 Supplemental Data

Western blots

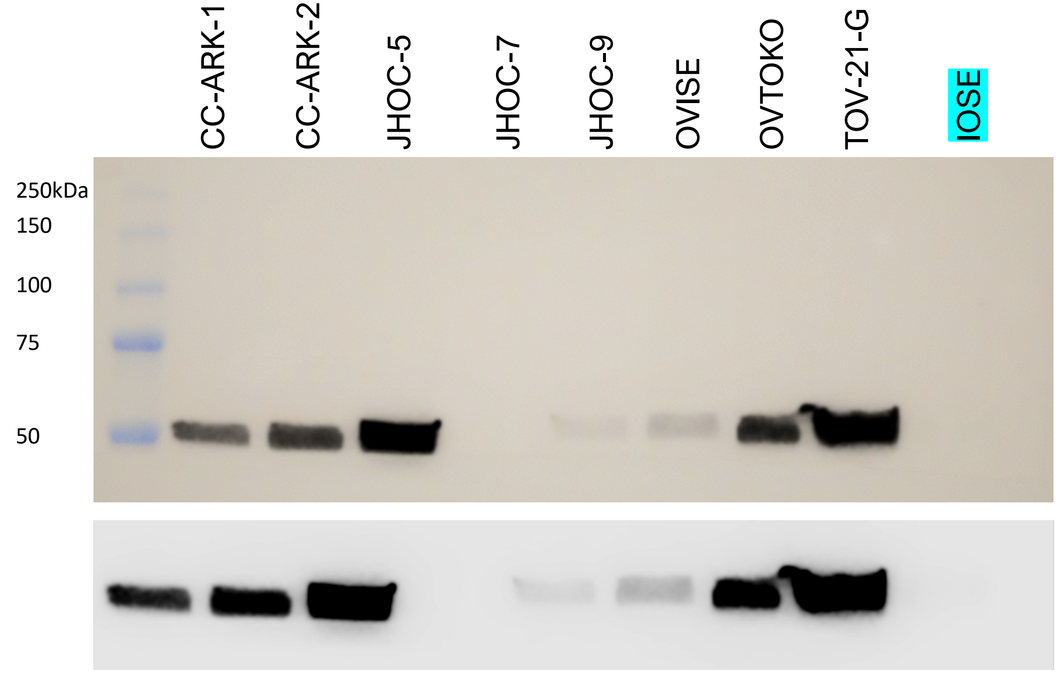

Class III β-Tubulin (OCCCs)

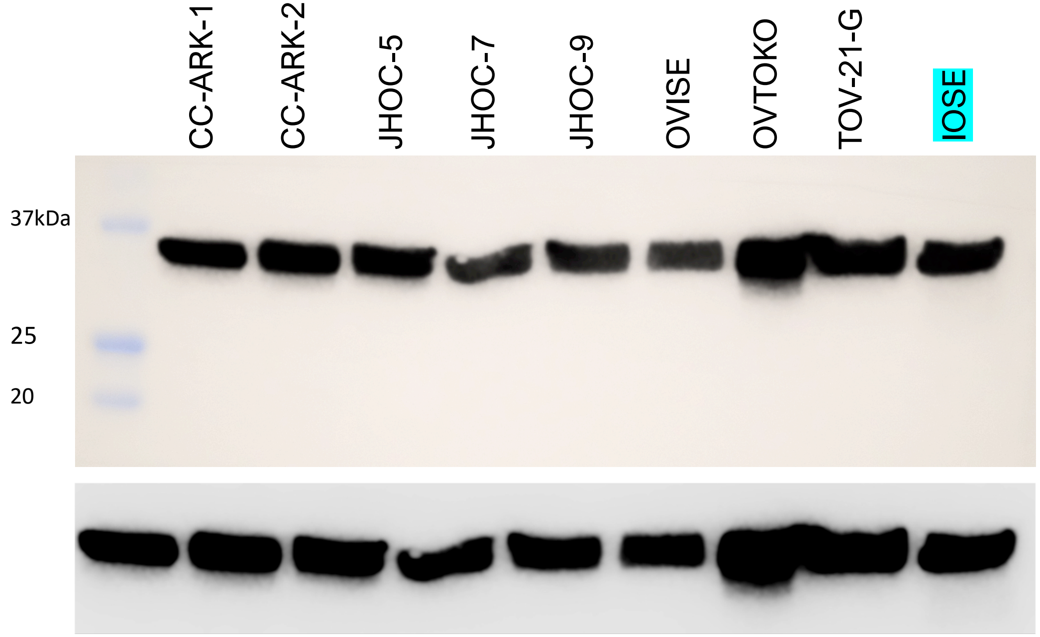

GAPDH (OCCCs)

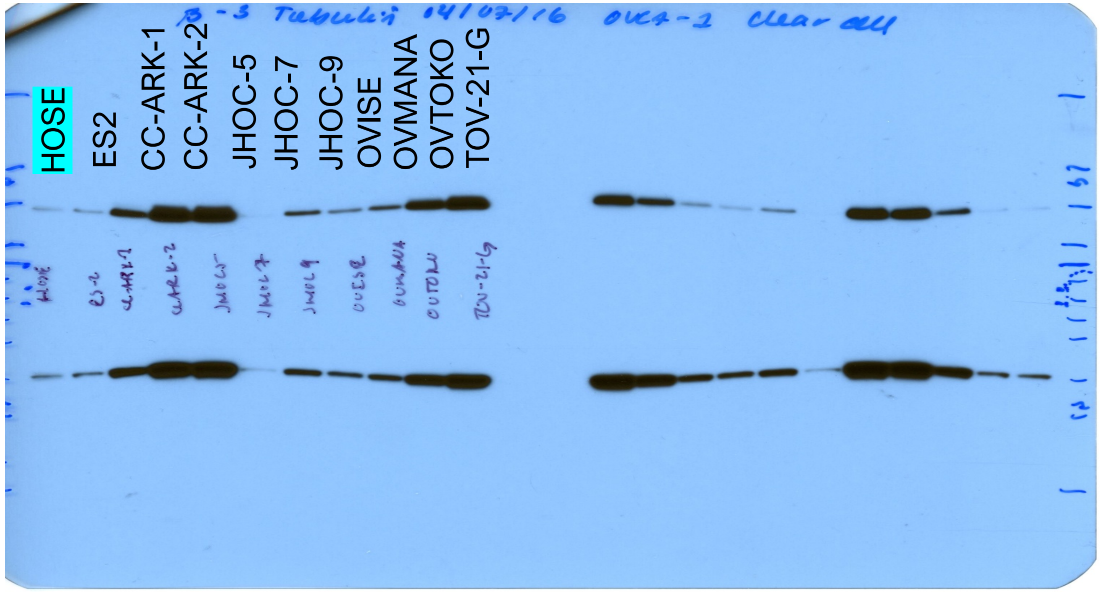

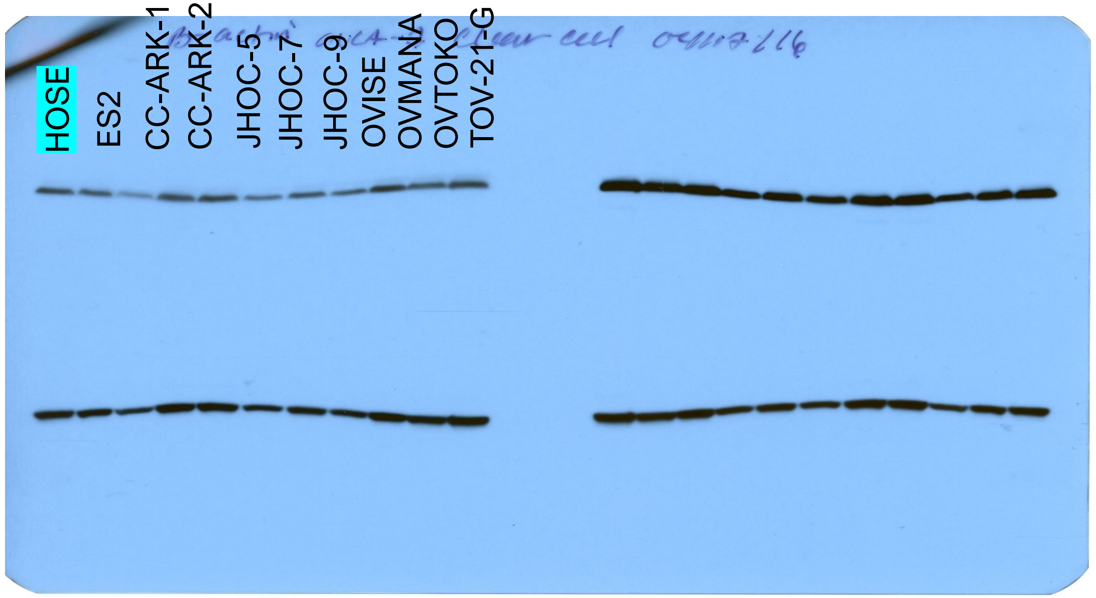

Class III Beta Tubulin OSC

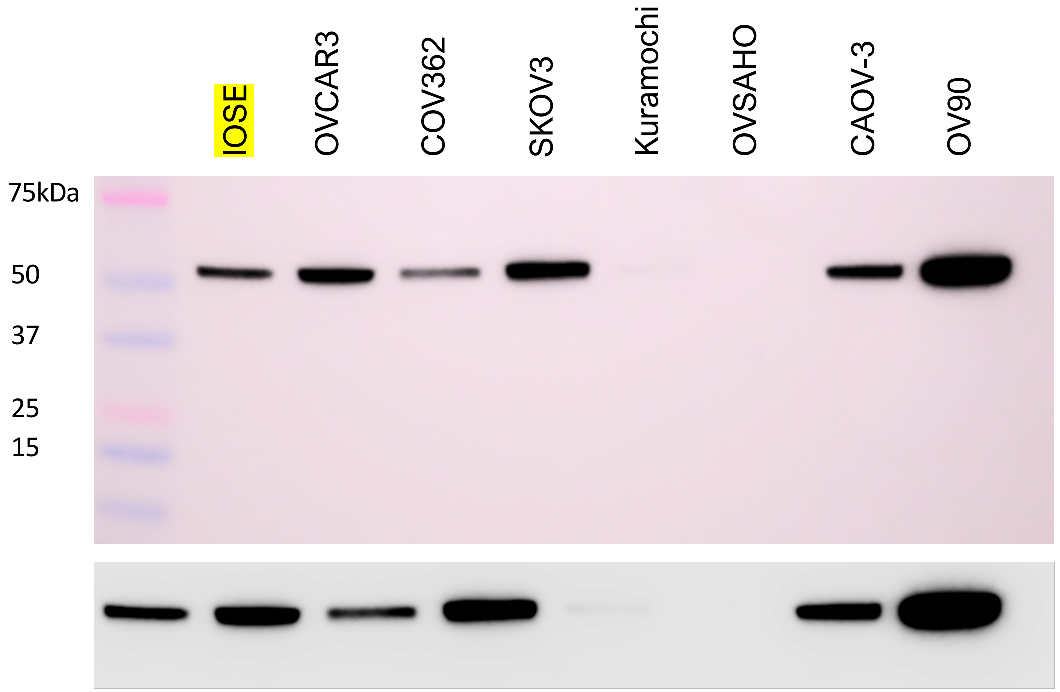

Class III β-Tubulin (OSCs)

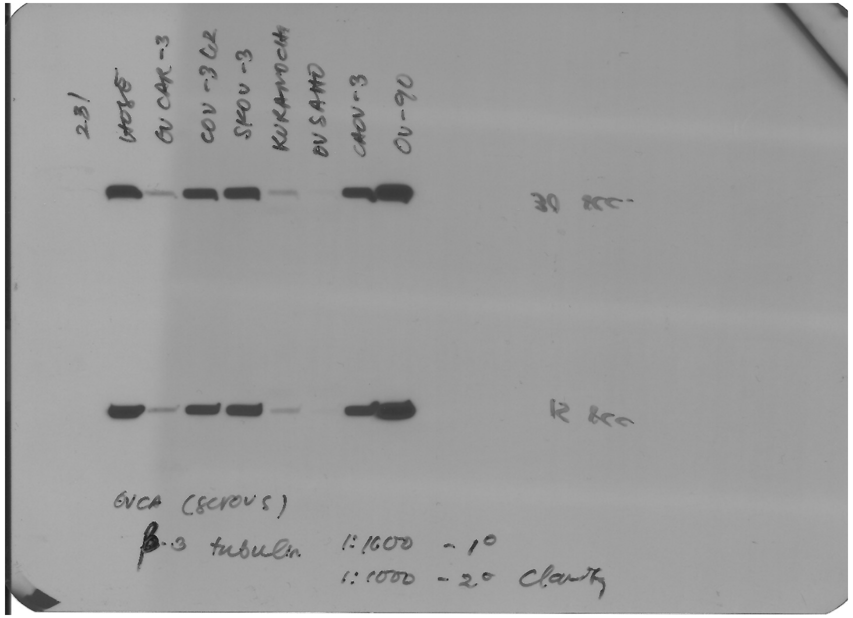

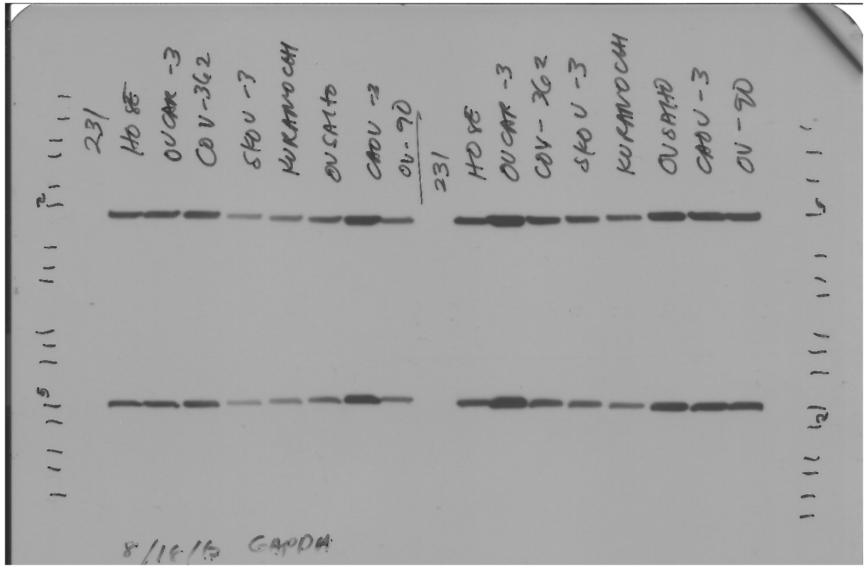

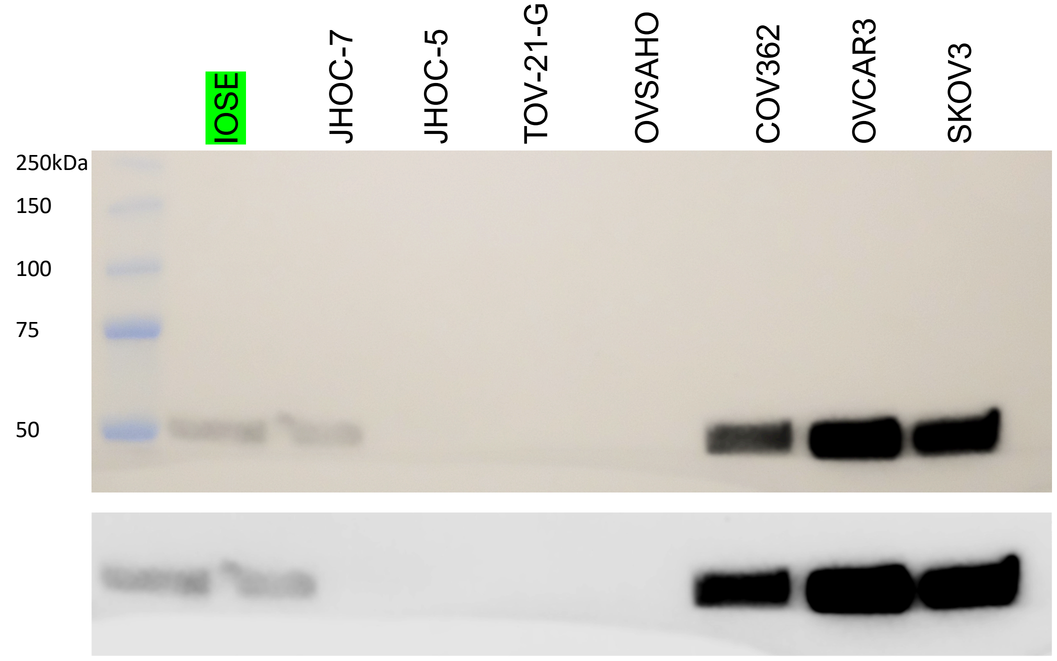

Detyrosinated Tubulin

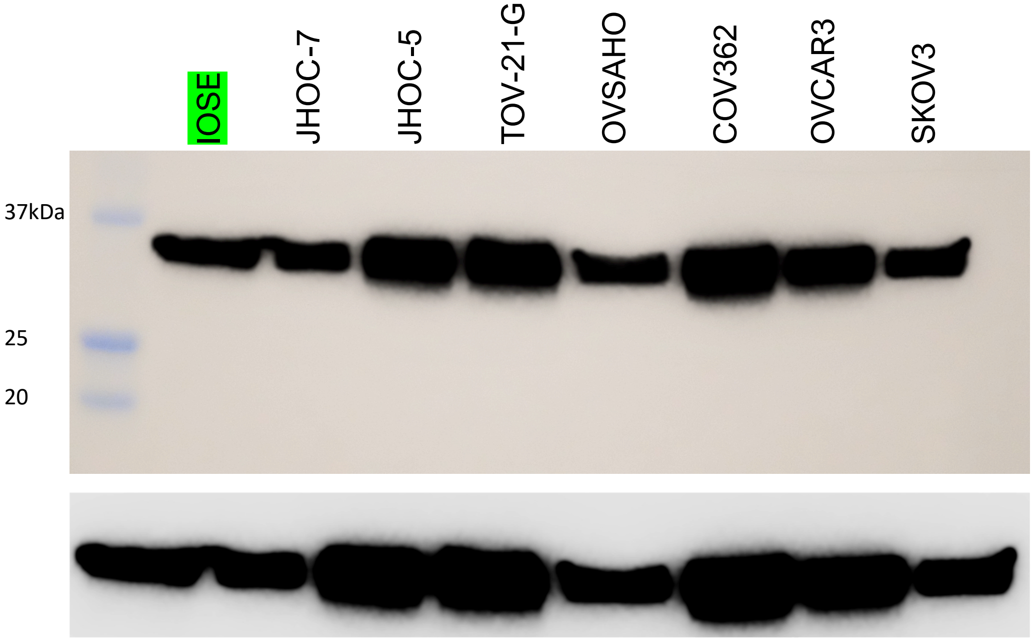

GAPDH Ctrl

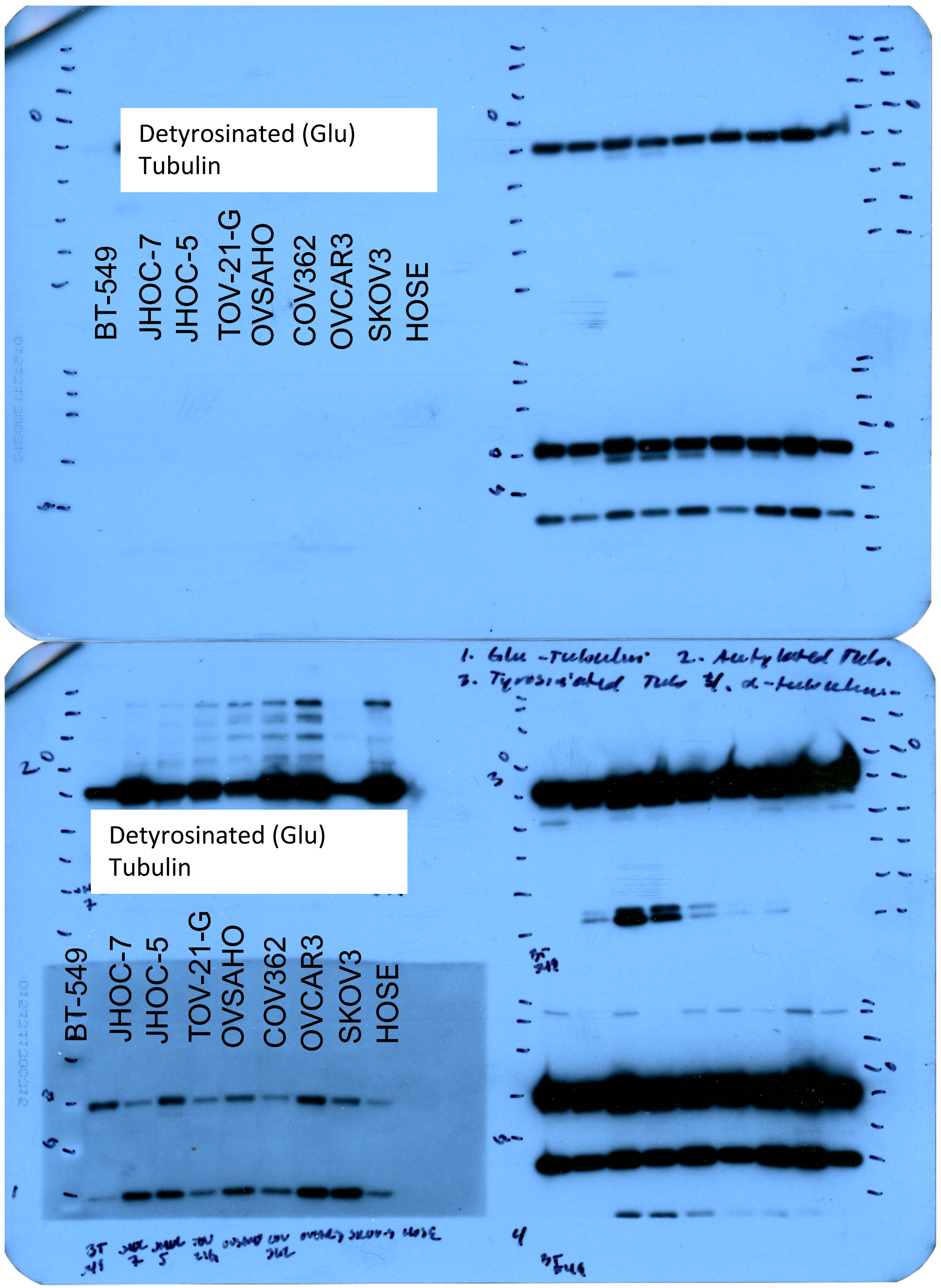

Short Exposure

Long Exposure

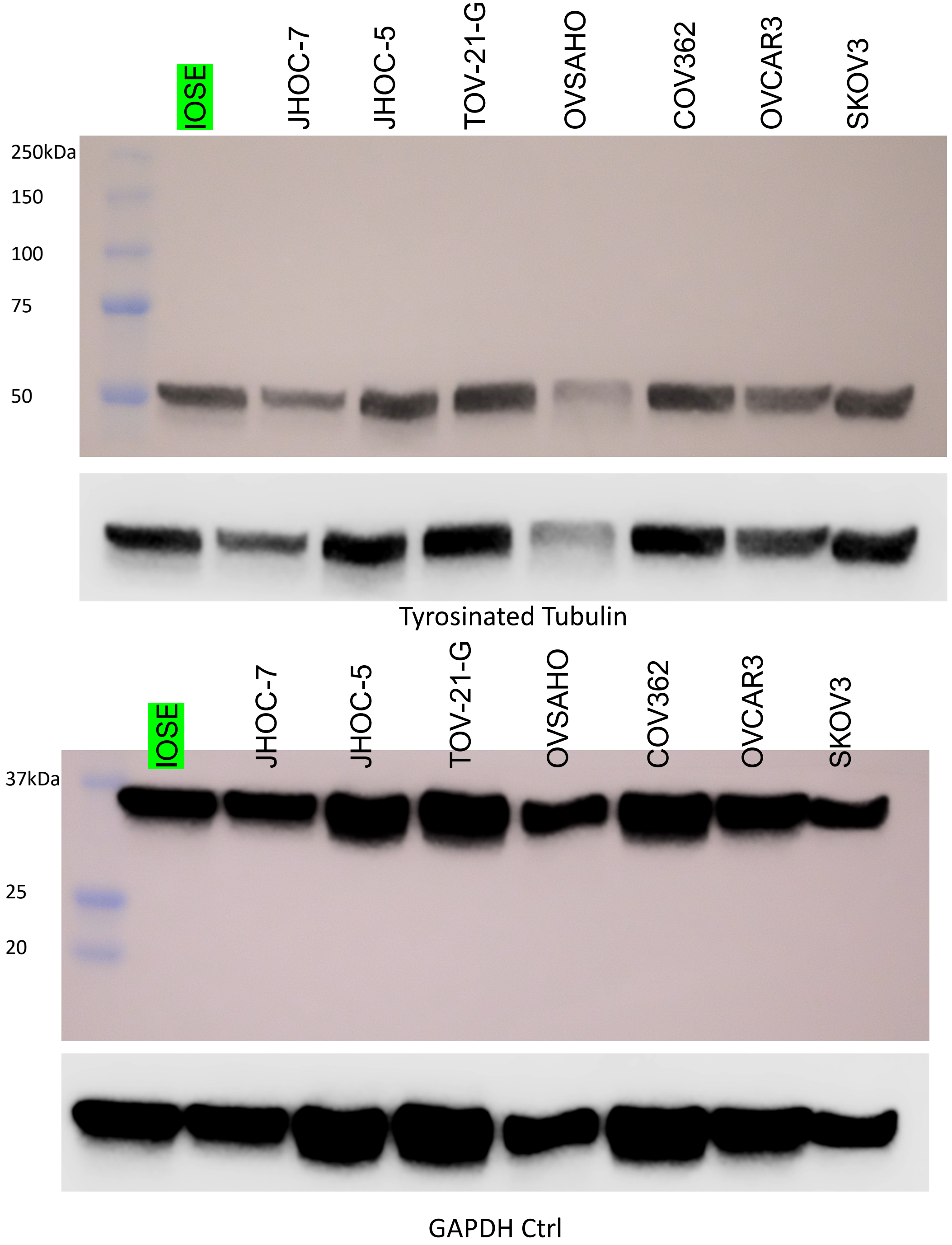

IOSE
JHOC-7
JHOC-5
TOV-21-G
OVSAHO
COV362
OVCAR3
SKOV3
250kDa
150
100
75
50
Tyrosinated Tubulin
IOSE
JHOC-7
JHOC-5
TOV-21-G
OVSAHO
COV362
OVCAR3
SKOV3
37kDa
25
20
GAPDH Ctrl

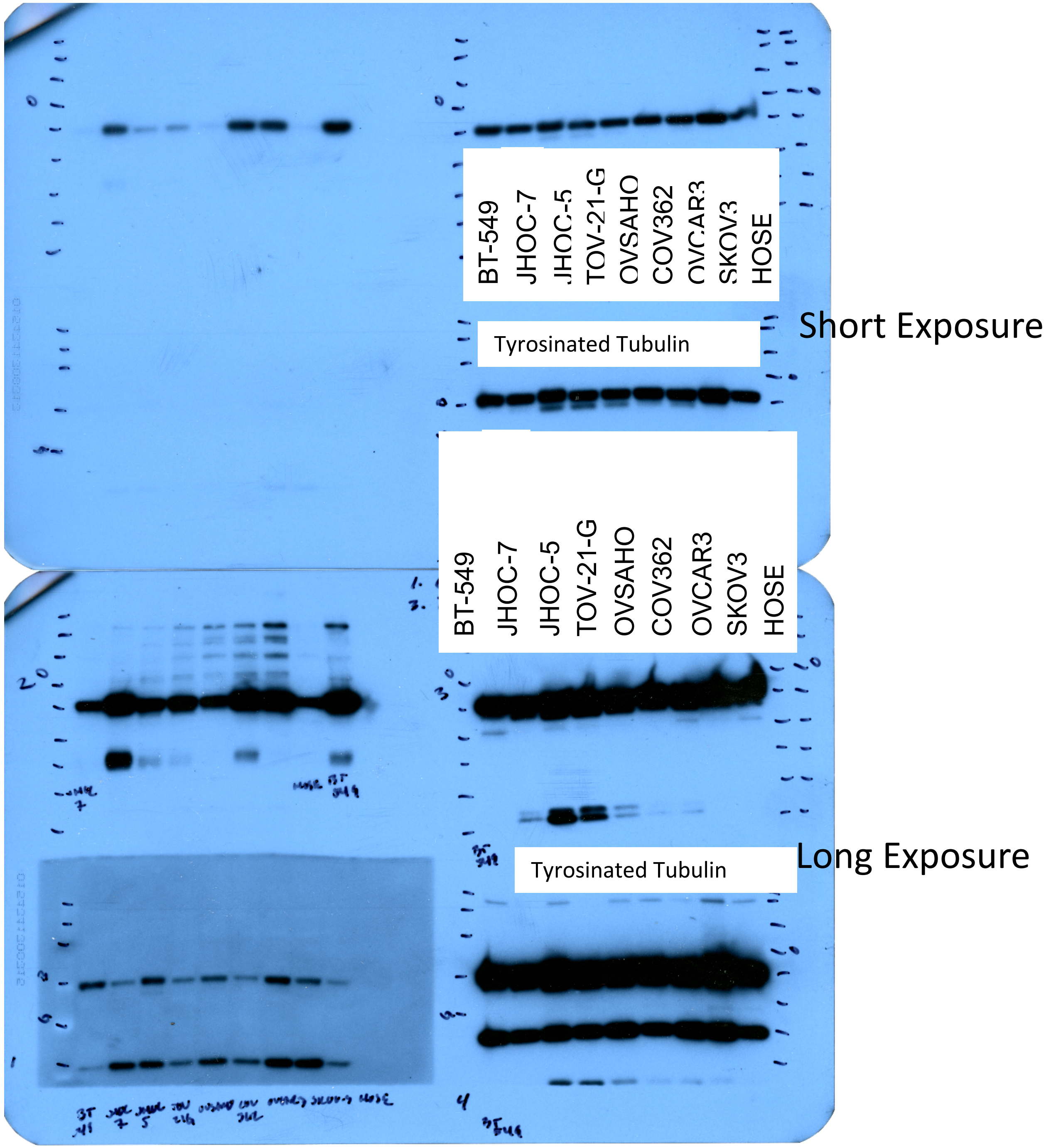

BT-549
JHOC-7
JHOC-5
TOV-21-G
OVSAHO
COV362
OVCAR3
SKOV3
HOSE
Tyrosinated Tubulin
Short Exposure
BT-549
JHOC-7
JHOC-5
TOV-21-G
OVSAHO
COV362
OVCAR3
SKOV3
HOSE
Tyrosinated Tubulin
Long Exposure

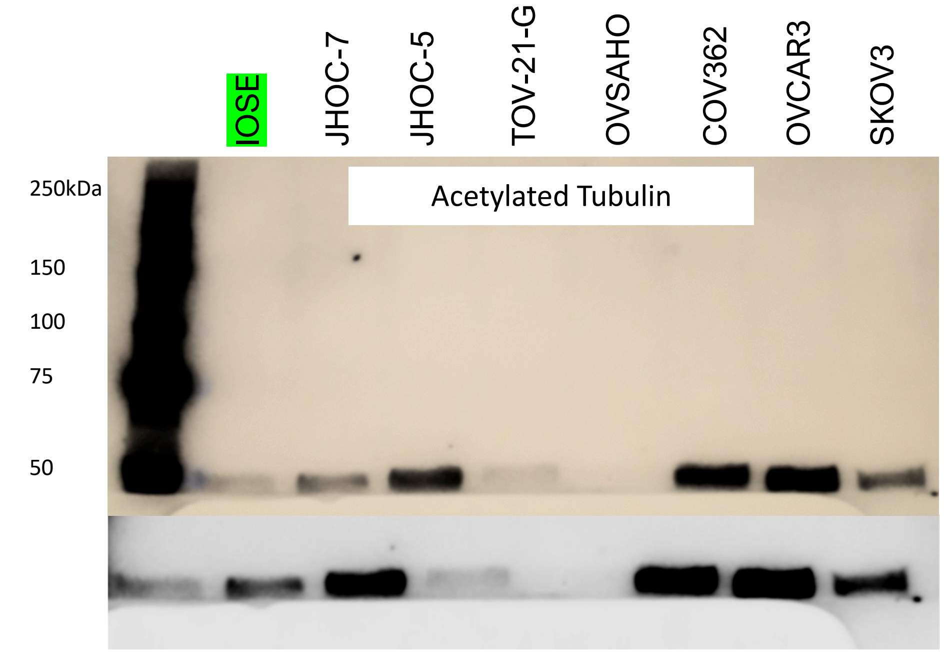
IOSE
JHOC-7
JHOC-5
TOV-21-G
OVSAHO
COV362
OVCAR3
SKOV3
Acetylated Tubulin
250kDa
150
100
75
50

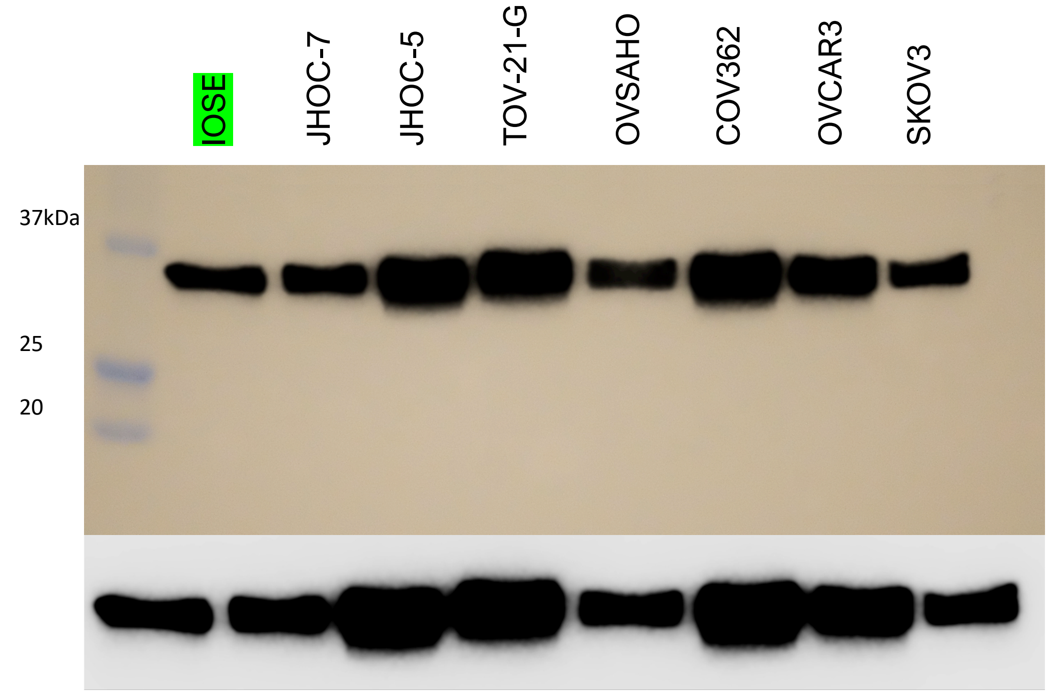
IOSE
JHOC-7
JHOC-5
TOV-21-G
OVSAHO
COV362
OVCAR3
SKOV3
37kDa
25
20

GAPDH Ctrl

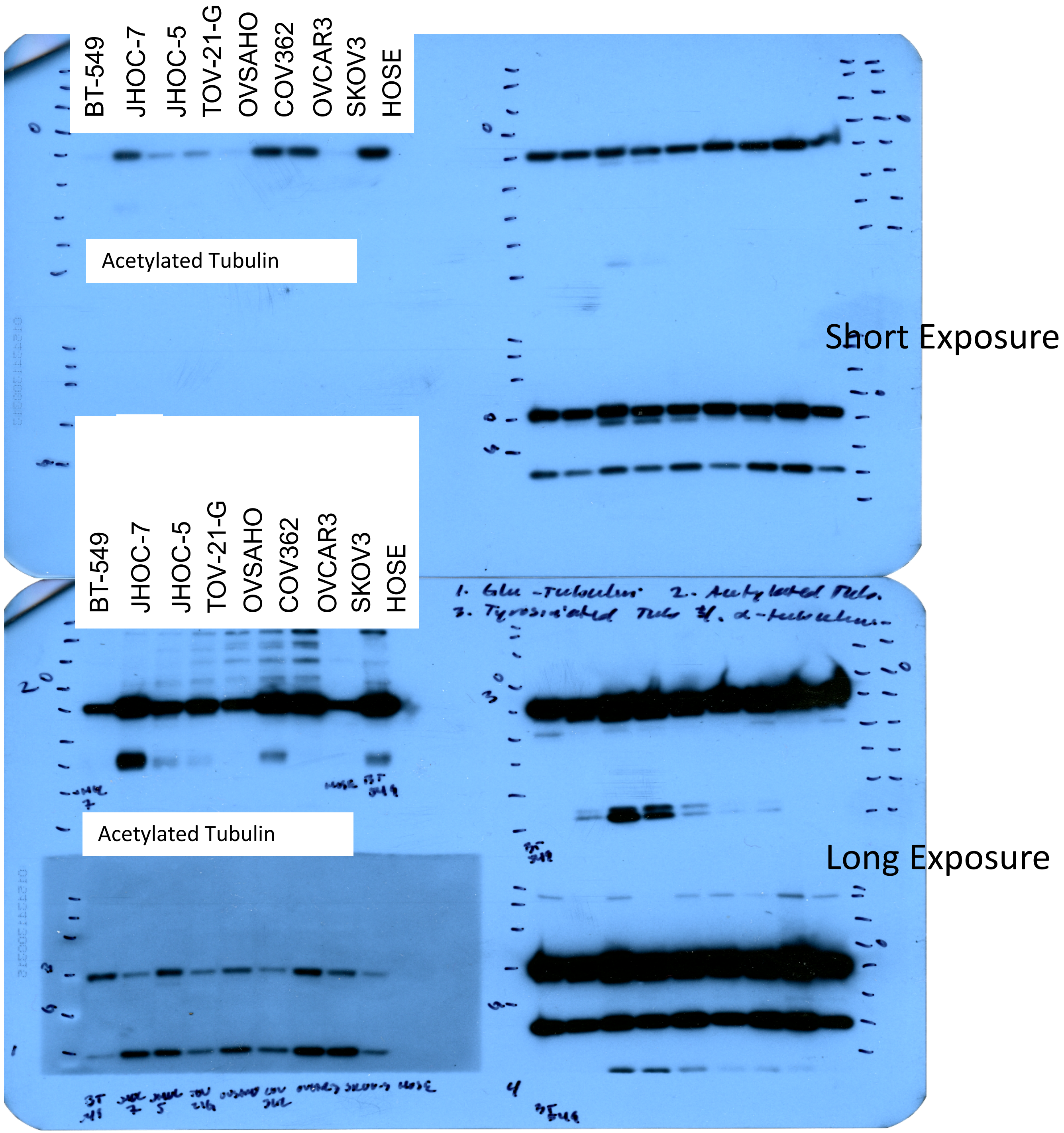
BT-549
JHOC-7
JHOC-5
TOV-21-G
OVSAHO
COV362
OVCAR3
SKOV3
HOSE
Acetylated Tubulin
Short Exposure
BT-549
JHOC-7
JHOC-5
TOV-21-G
OVSAHO
COV362
OVCAR3
SKOV3
HOSE
Acetylated Tubulin
Long Exposure

Figure 4 Supplemental Data

Microtentacle Length and Number. Analysis of microtentacle length and number after treatment with vehicle, paclitaxel, ixabepilone, colchicine or vinblastine.

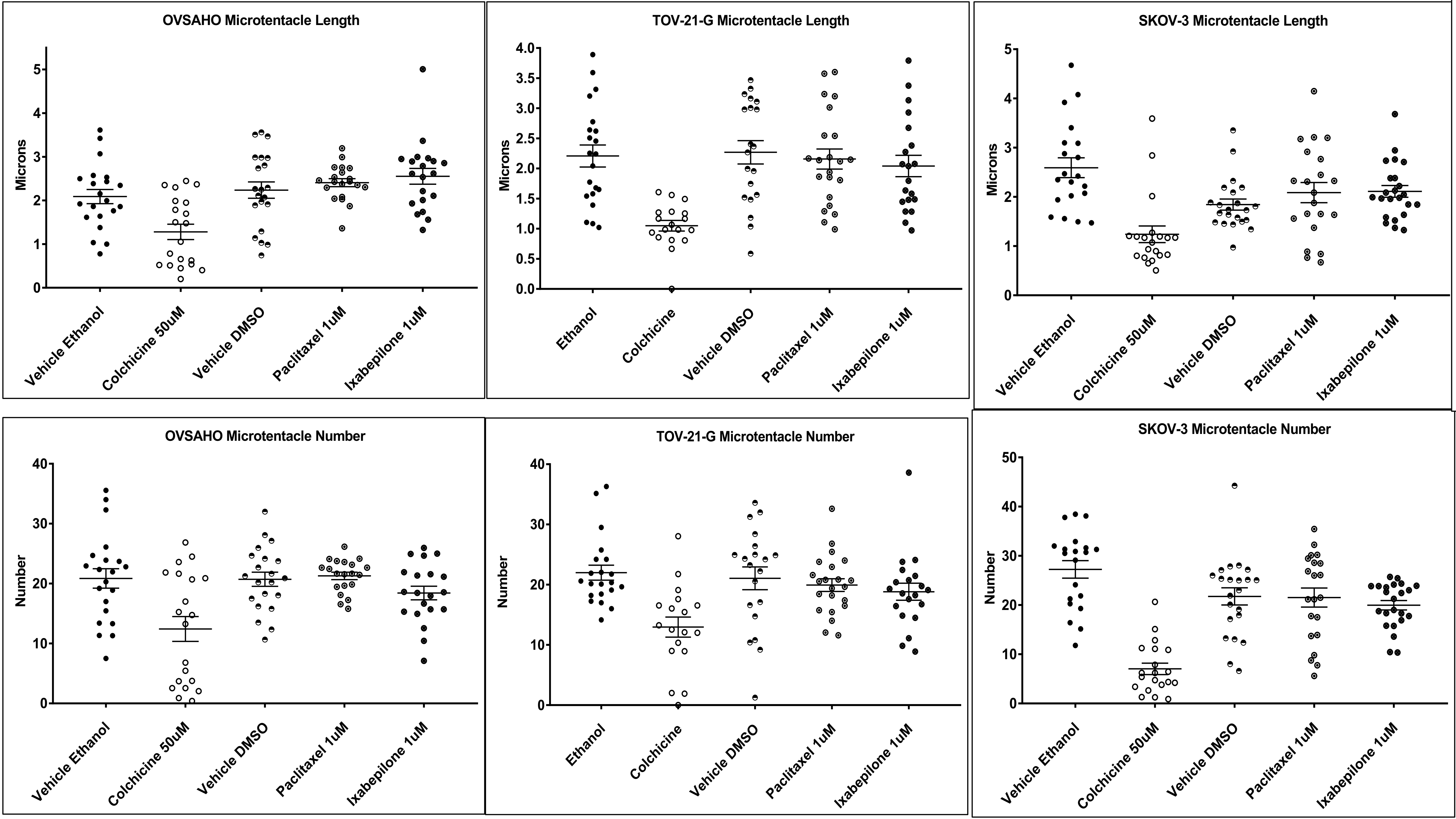
OVSAHO Microtentacle Length
Microns
Vehicle Ethanol
Colchicine 50uM
Vehicle DMSO
Paclitaxel 1uM
Ixabepilone 1uM
TOV-21-G Microtentacle Length
Microns
Ethanol
Colchicine
Vehicle DMSO
Paclitaxel 1uM
Ixabepilone 1uM
SKOV-3 Microtentacle Length
Microns
Vehicle Ethanol
Colchicine 50uM
Vehicle DMSO
Paclitaxel 1uM
Ixabepilone 1uM
OVSAHO Microtentacle Number
Number
Vehicle Ethanol
Colchicine 50uM
Vehicle DMSO
Paclitaxel 1uM
Ixabepilone 1uM
TOV-21-G Microtentacle Number
Number
Ethanol
Colchicine
Vehicle DMSO
Paclitaxel 1uM
Ixabepilone 1uM
SKOV-3 Microtentacle Number
Number
Vehicle Ethanol
Colchicine 50uM
Vehicle DMSO
Paclitaxel 1uM
Ixabepilone 1uM

Figure 7 Supplemental Data

Western Blots

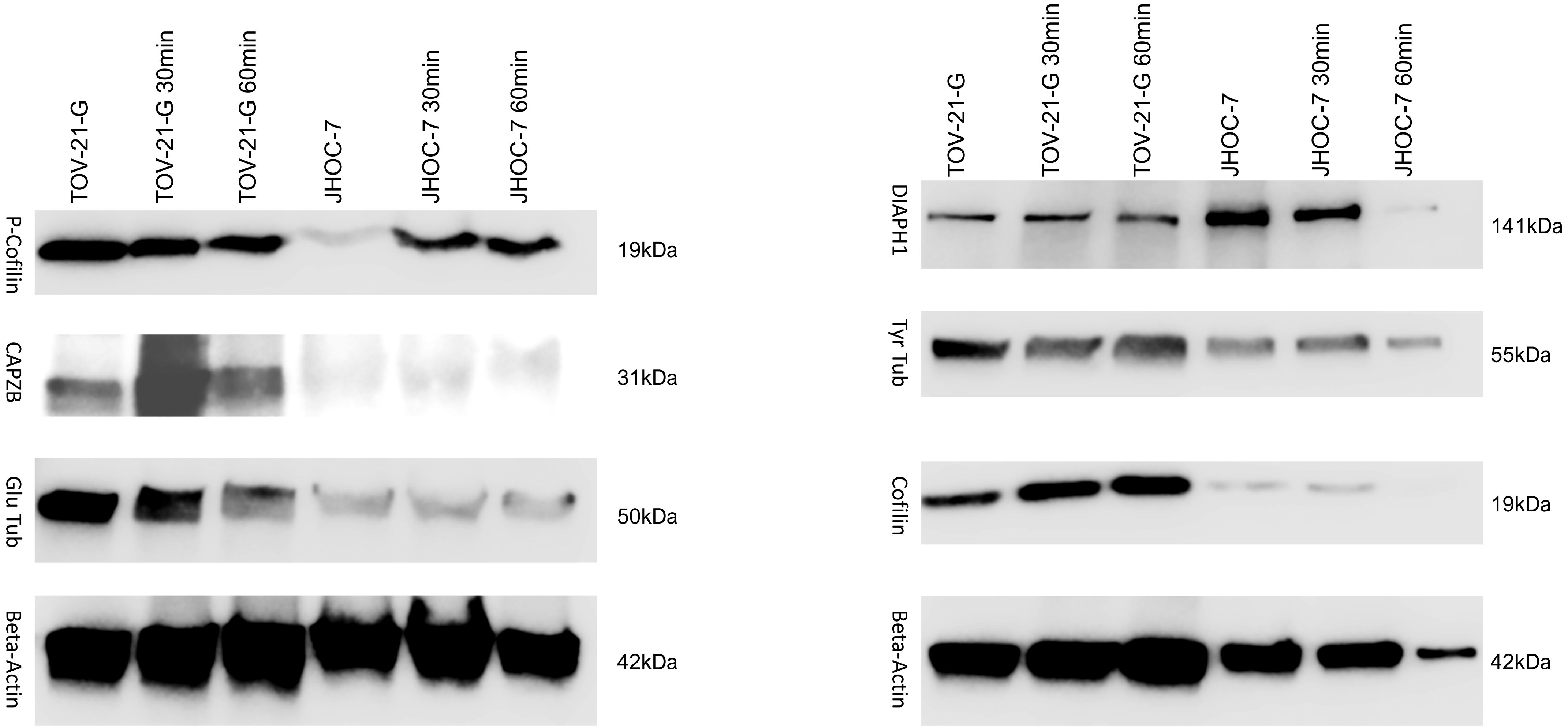
TOV-21-G
TOV-21-G 30min
TOV-21-G 60min
JHOC-7
JHOC-7 30min
JHOC-7 60min
P-Cofilin
19kDa
CAPZB
31kDa
Glu Tub
50kDa
Beta-Actin
42kDa
TOV-21-G
TOV-21-G 30min
TOV-21-G 60min
JHOC-7
JHOC-7 30min
JHOC-7 60min
DIAPH1
141kDa
Tyr Tub
55kDa
Cofilin
19kDa
Beta-Actin
42kDa

Figure 7

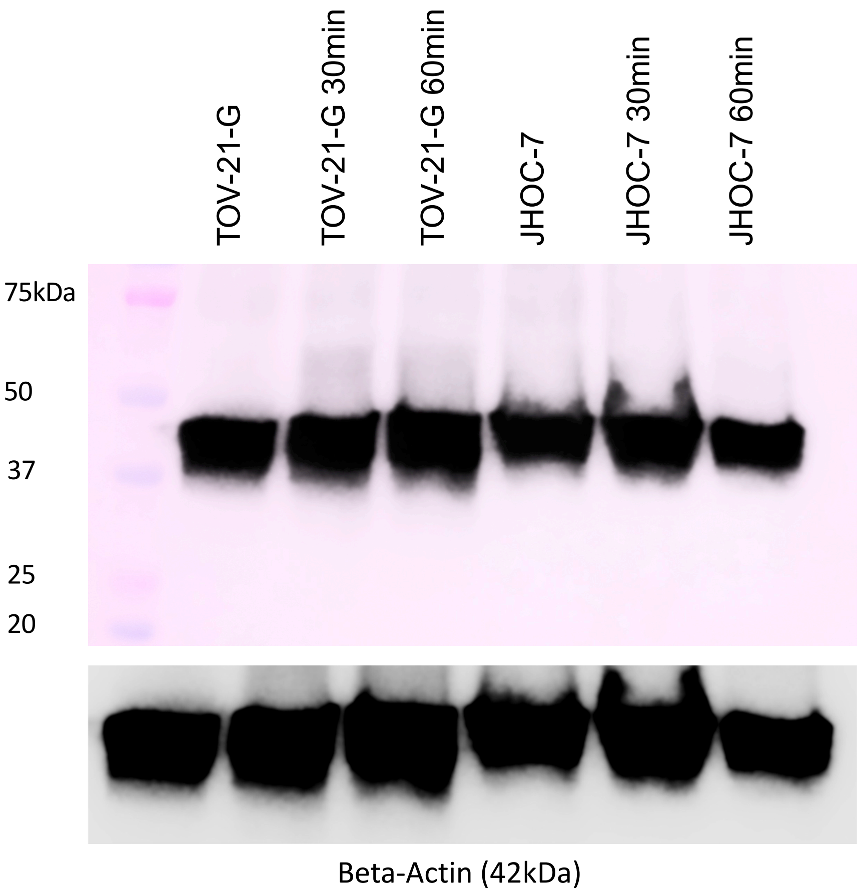

TOV-21-G
TOV-21-G 30min
TOV-21-G 60min
JHOC-7
JHOC-7 30min
JHOC-7 60min
75kDa
50
37
25
20
Beta-Actin (42kDa)

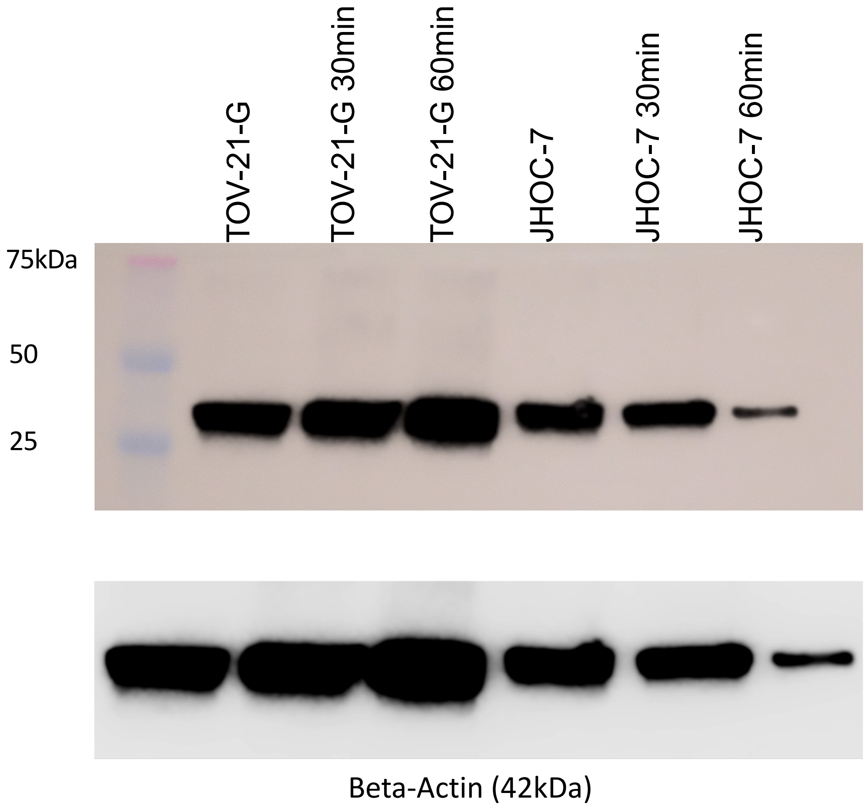

TOV-21-G
TOV-21-G 30min
TOV-21-G 60min
JHOC-7
JHOC-7 30min
JHOC-7 60min
75kDa
50
25
Beta-Actin (42kDa)

Figure 7

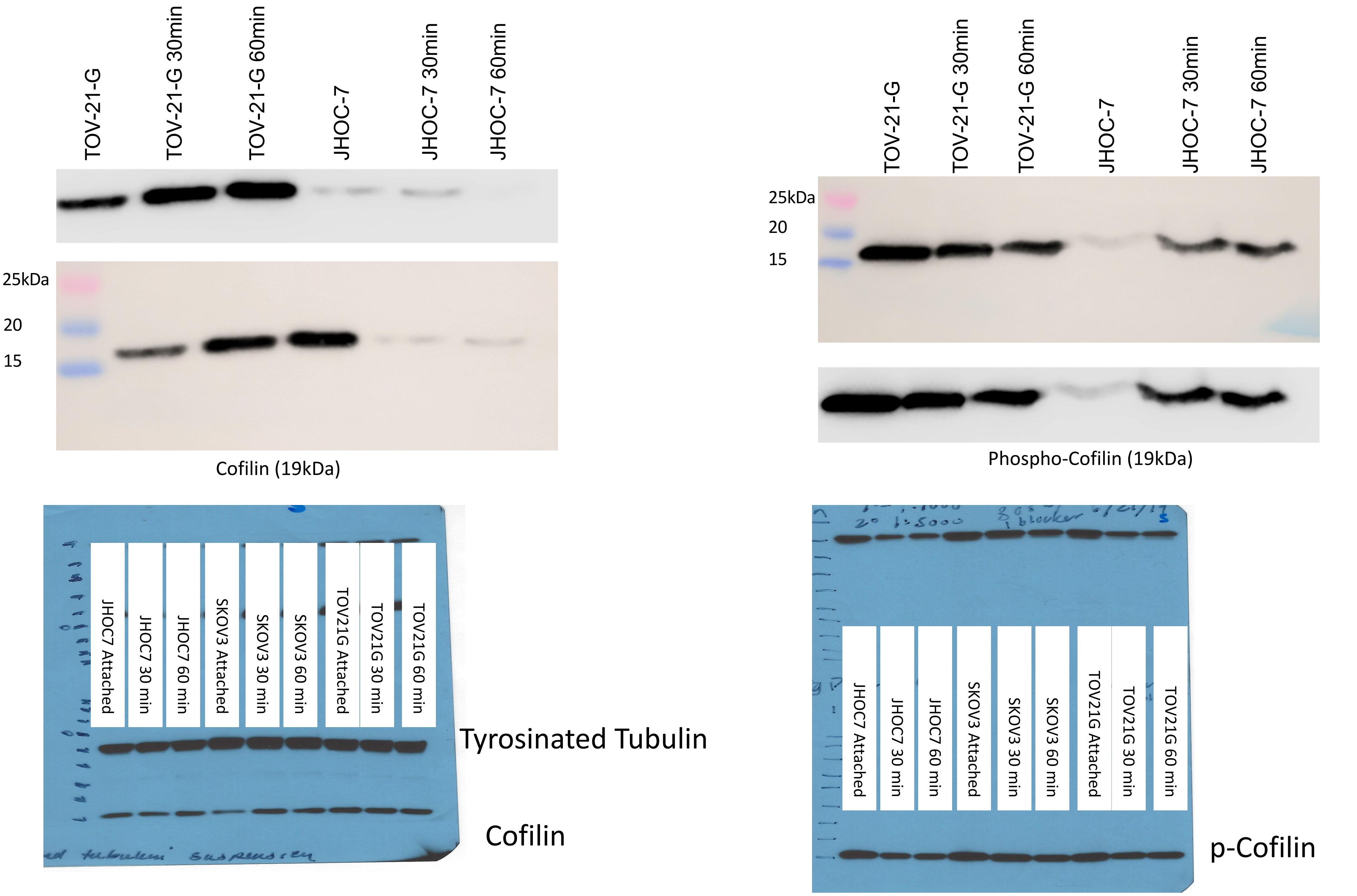
TOV-21-G
TOV-21-G 30min
TOV-21-G 60min
JHOC-7
JHOC-7 30min
JHOC-7 60min
25kDa
20
15
Cofilin (19kDa)
TOV-21-G
TOV-21-G 30min
TOV-21-G 60min
JHOC-7
JHOC-7 30min
JHOC-7 60min
25kDa
20
15
Phospho-Cofilin (19kDa)
JHOC7 Attached
JHOC7 30 min
JHOC7 60 min
SKOV3 Attached
SKOV3 30 min
SKOV3 60 min
TOV21G Attached
TOV21G 30 min
TOV21G 60 min
Tyrosinated Tubulin
Cofilin
JHOC7 Attached
JHOC7 30 min
JHOC7 60 min
SKOV3 Attached
SKOV3 30 min
SKOV3 60 min
TOV21G Attached
TOV21G 30 min
TOV21G 60 min
p-Cofilin

Figure 7

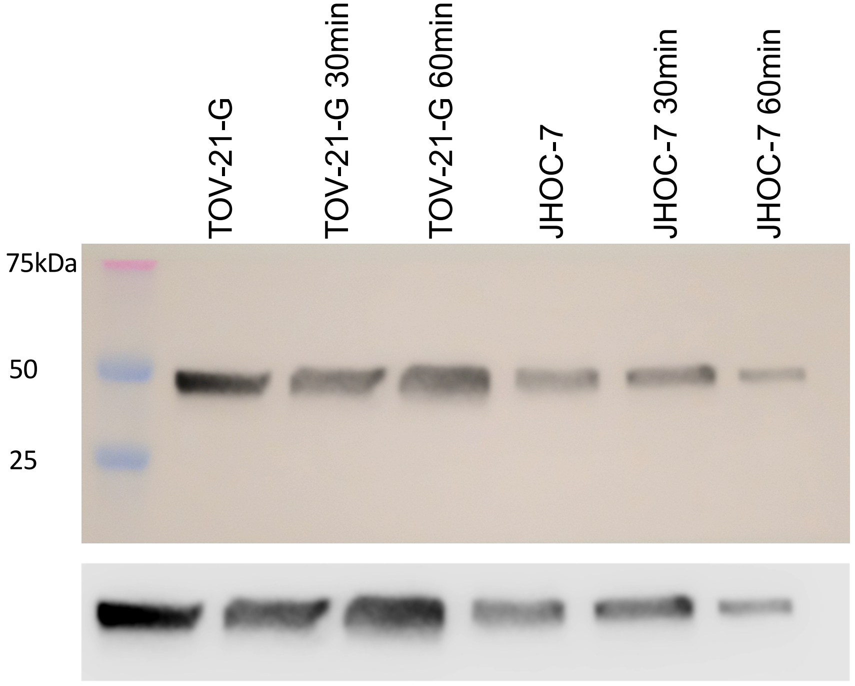

Tyrosinated Tubulin (55kDa)

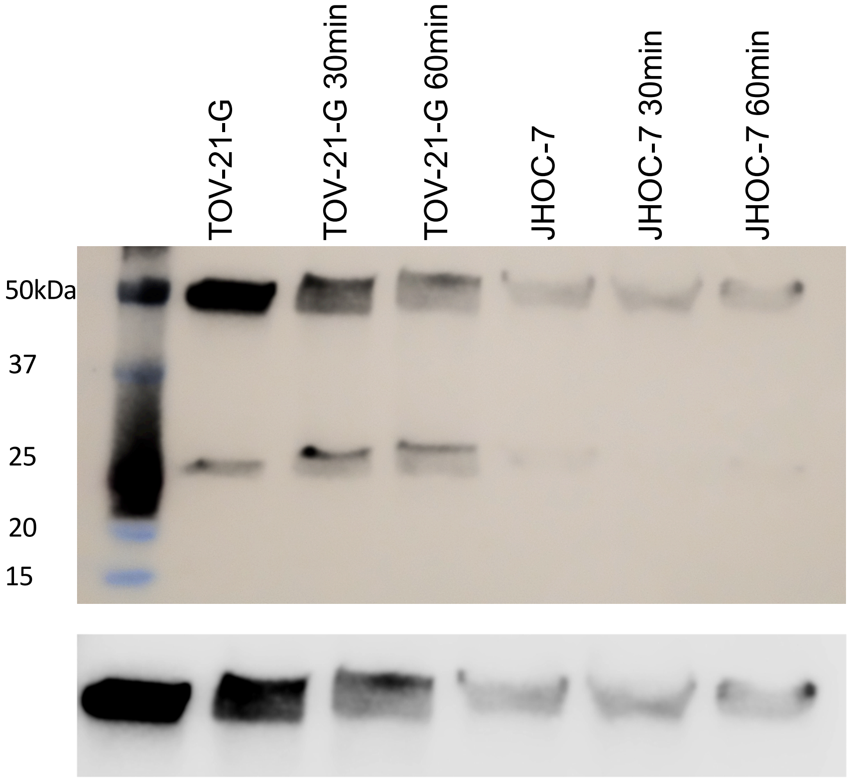

Detyrosinated Tubulin (50kDa)

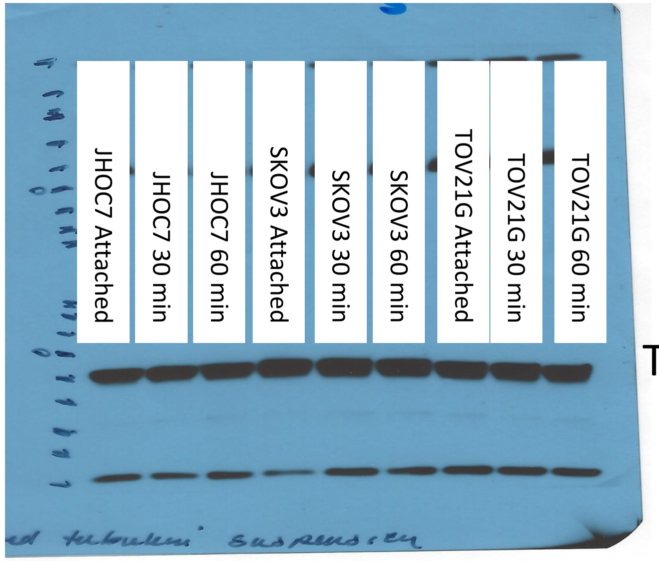

Tyrosinated Tubulin

Cofilin

Figure 7

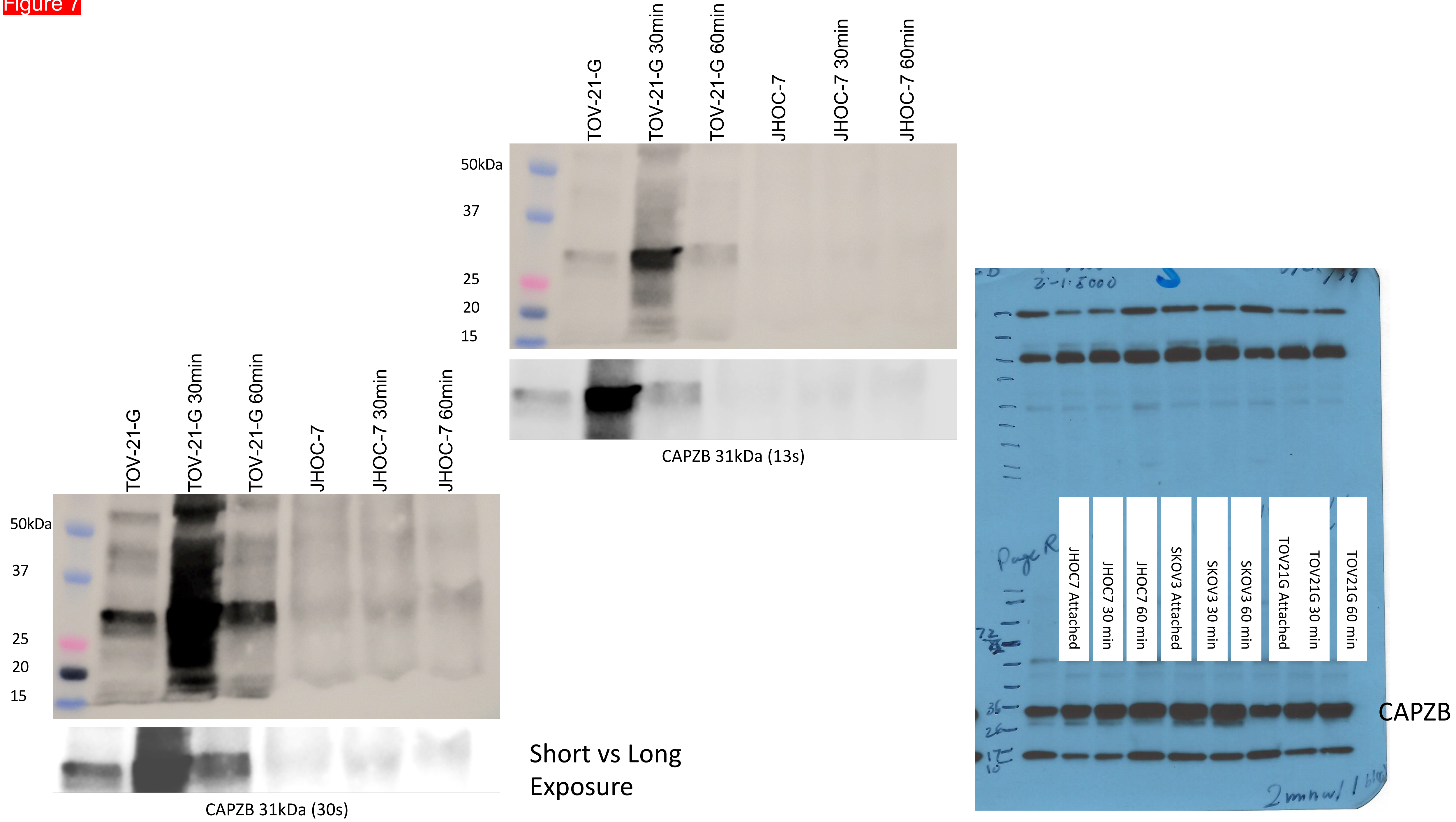
TOV-21-G
TOV-21-G 30min
TOV-21-G 60min
JHOC-7
JHOC-7 30min
JHOC-7 60min
50kDa
37
25
20
15
CAPZB 31kDa (13s)
TOV-21-G
TOV-21-G 30min
TOV-21-G 60min
JHOC-7
JHOC-7 30min
JHOC-7 60min
50kDa
37
25
20
15
CAPZB 31kDa (30s)
Short vs Long Exposure
JHOC7 Attached
JHOC7 30 min
JHOC7 60 min
SKOV3 Attached
SKOV3 30 min
SKOV3 60 min
TOV21G Attached
TOV21G 30 min
TOV21G 60 min
CAPZB

Figure 7

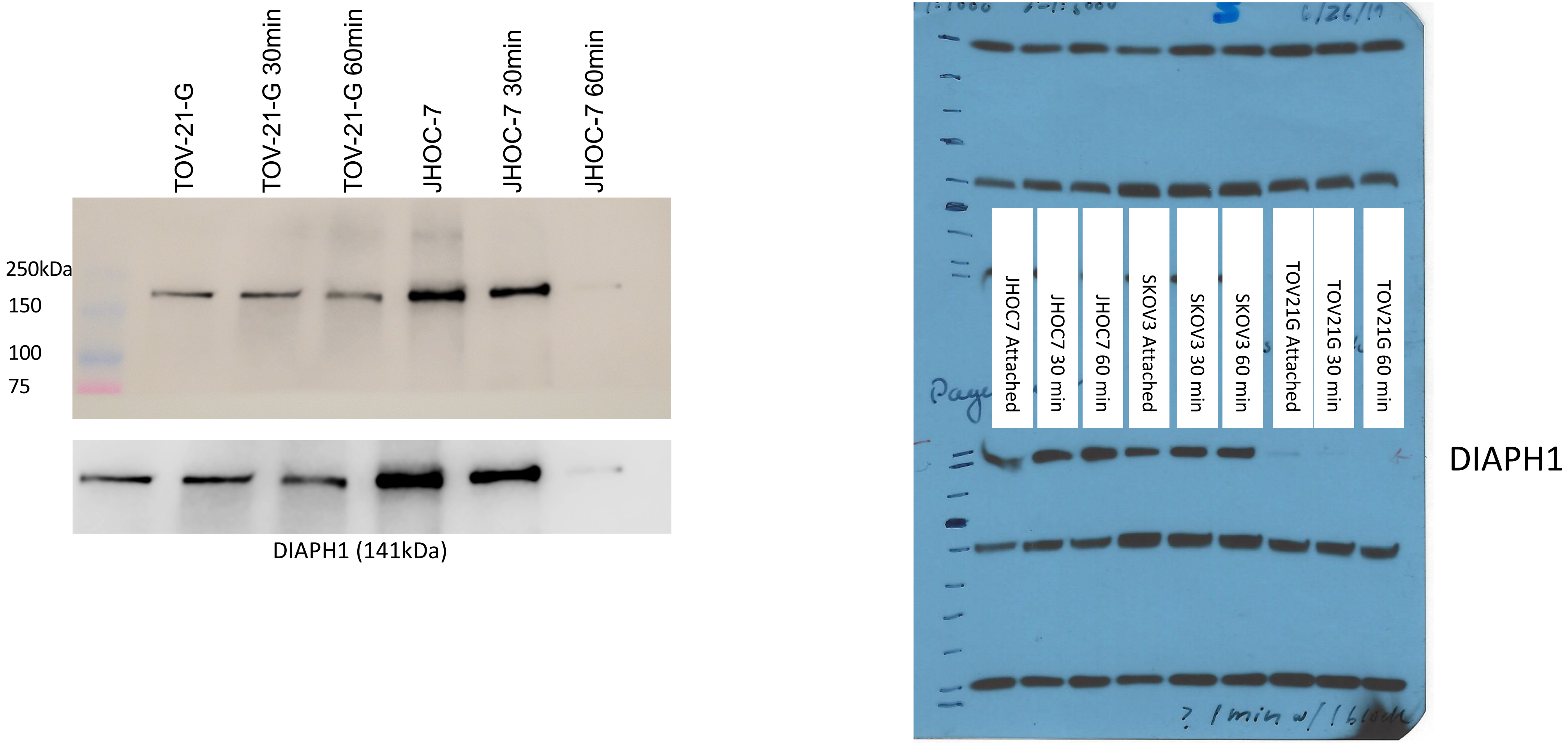

TOV-21-G
TOV-21-G 30min
TOV-21-G 60min
JHOC-7
JHOC-7 30min
JHOC-7 60min
250kDa
150
100
75
DIAPH1 (141kDa)
JHOC7 Attached
JHOC7 30 min
JHOC7 60 min
SKOV3 Attached
SKOV3 30 min
SKOV3 60 min
TOV21G Attached
TOV21G 30 min
TOV21G 60 min
DIAPH1

Supplemental Table S1. Characteristics of cell lines. *NR=not reported*

CELL LINE	AGE (years)	STAGE	TREATMENT STATUS	SOURCE
Clear Cell				
CC-ARK1	42	II	chemo-naÏve	Roque DM, Bellone S, Buza N, et al. Class III β-tubulin overexpression in ovarian clear cell and serous carcinoma as a maker for poor overall survival after platinum/taxane chemotherapy and sensitivity to patupilone. Am J Obstet Gynecol. 2013 Jul;209(1):62.e1-9
CC-ARK2	32	IC	chemo-naÏve	Roque DM, Bellone S, Buza N, et al. Class III β-tubulin overexpression in ovarian clear cell and serous carcinoma as a maker for poor overall survival after platinum/taxane chemotherapy and sensitivity to patupilone. Am J Obstet Gynecol. 2013 Jul;209(1):62.e1-9
TOV21G	62	III	chemo-naÏve	Provencher, D. M. et al. Characterization of four novel epithelial ovarian cancer cell lines. In Vitro Cell. Dev. Biol. Anim. 36, 357–361 (2000).
JHOC-5	54	IIC	NR	Yamada, K. et al. Establishment and characterization of cell lines derived from serous adenocarcinoma (JHOS-2) and clear cell adenocarcinoma (JHOC-5, JHOC-6) of human ovary. Hum. Cell 12, 131–138 (1999).
JHOC-7	NR	NR	NR	Not commercially reported
OVTOKO	78	IIIB	recurrent	Gorai, I. et al. Establishment and characterization of two human ovarian clear cell adenocarcinoma lines from metastatic lesions with different properties. Gynecol. Oncol. 57, 33–46 (1995).
OVISE	40	IIB	recurrent	Gorai, I. et al. Establishment and characterization of two human ovarian clear cell adenocarcinoma lines from metastatic lesions with different properties. Gynecol. Oncol. 57, 33–46 (1995).
OVMANA	NR	NR	recurrent	Yanagibashi, T. et al. Complexity of expression of the intermediate filaments of six new human ovarian carcinoma cell lines: new expression of cytokeratin 20. Br. J. Cancer 76, 829–835 (1997).
Serous				
Kuramochi	NR	NR	NR	Motoyama T. Quantitative analysis on *in vitro* drug sensitivity of cultured human ovarian cancer cell lines. Nihon Sanka Fujinka Gakkai Zasshi 1982; 34(3):308-14 (Japanese)..
OVSAHO	NR	≥ III	recurrent	Yanagibashi, T. et al. Complexity of expression of the intermediate filaments of six new human ovarian carcinoma cell lines: new expression of cytokeratin 20. Br. J. Cancer 76, 829–835 (1997).
CaOV3	54	NR	NR	Fogh J, Fogh JM, Orfeo T. One hundred and twenty-seven cultured human tumor cell lines producing tumors in nude mice. Journal of the National Cancer Institute 1977; 59(1):221-6. See also https://www.mskcc.org/research-advantage/support/technology/tangible-material/caov-3-human-ovarian-cell-line
COV362	NR	IV	NR	van den Berg-Bakker CAM, Hagemeijer A, Franken-Postma EM, et al. Establishment and characterization of 7 ovarian carcinoma cell lines and one granulosa tumor cell line: Growth features and cytogenetics. Experimental Cancer 1993; 53(4):613-20.
SKOV3	64	NR	NR	Fogh J, Fogh JM, Orfeo T. One hundred and twenty-seven cultured human tumor cell lines producing tumors in nude mice. Journal of the National Cancer Institute 1977; 59(1):221-6.
OVCAR3	60	NR	progressive	Hamilton TC, Young RC, McKoy WM, et al. Characterization of a human ovarian carcinoma cell line (NIH:OVCAR-3) with androgen and estrogen receptors. Cancer Res 1983;43(11):5379-89.
OV90	64	IIIC	chemo-naÏve	Provencher DM, Lounis H, Champoux L, et al. Characterization of four novel epithelial ovarian cancer cell lines. In Vitro Cell Dev Biol Anim 2000;36(6):357-61.
Clear cell-like				
ES2	47	NR	NR	Kwok ALM, Wong OG-W, Wong ESY, et al. Caution over use of ES2 as a model of ovarian clear cell carcinoma. J Clin Pathol 2014;67(10):921-2.
